# Ketamine does not rescue plaque load or gap detection in the 5XFAD mouse model of Alzheimer's disease

**DOI:** 10.3389/fnagi.2025.1505908

**Published:** 2025-02-03

**Authors:** Alexa L. Wright, Aldis P. Weible, Olivia B. Estes, Michael Wehr

**Affiliations:** Department of Psychology, Institute of Neuroscience, University of Oregon, Eugene, OR, United States

**Keywords:** gap detection, behavior, ketamine, biomarkers, auditory cortex, psychoplastogens, 5xFAD

## Abstract

Ketamine has received growing attention for its effects on neuroplasticity and neuroinflammation, and as a treatment for depression and other mental health disorders. Recent evidence suggests that early sensory and behavioral deficits in Alzheimer's disease could be caused by synaptic disruption that occurs before irreversible neuropathology. This raises the possibility that ketamine could slow down or prevent network disruption and the ensuing sensory and behavioral deficits in Alzheimer's. Here we tested this idea in the 5XFAD mouse model of Alzheimer's, using either an acute single injection of ketamine, or chronic daily injections over 15 weeks. We tested the effects of ketamine on both amyloid plaque load and on a behavioral auditory gap detection task that is an early Alzheimer's biomarker in both mice and humans. We found that ketamine had no effect on plaque load, nor any effect on gap detection, for either acute or chronic dosing. Chronic ketamine facilitated startle responses specifically in 5XFAD mice, but this could simply be related to experience-dependent effects on stress or habituation rather than any rescue effect of ketamine on Alzheimer's-related deficits. We did find robust correlations between gap detection deficits and plaque load in auditory cortex and in the caudal pontine reticular nucleus, demonstrating that the behavioral deficits seen in 5XFAD mice are directly related to amyloid accumulation in these brain regions, and confirming the validity of gap detection as an early biomarker of Alzheimer's. Ketamine, however, had no effect on the strength of these correlations. We conclude that ketamine has no beneficial effect on the development of behavioral gap detection deficits or plaque load in the 5XFAD Alzheimer's mouse model, following either an acute single dose or a chronic daily dose regimen.

## Introduction

Neuropathology is a hallmark of Alzheimer's disease, but permanent damage such as amyloid plaques and neuronal death are not the first steps in disease progression. Sensory processing and behavioral deficits can occur much earlier, which has led to the hypothesis that these deficits could be caused by synaptic disruption that occurs before irreversible neuropathology (Selkoe, 2002; Holtzman et al., 2011; Mucke and Selkoe, 2012; Buskila et al., 2013; Weible et al., 2020; Forner et al., 2021; Weible and Wehr, 2022). This raises the possibility that therapeutic interventions that target synaptic or neuronal plasticity could slow down or prevent network disruption and the ensuing sensory and behavioral deficits.

Hallucinogens, including ketamine and psilocybin, have received growing attention for their ability to promote synaptic and neuronal plasticity, and have been recognized as remarkably effective treatments for depression and substance use disorder (Berman et al., 2000; Aleksandrova and Phillips, 2021). It remains unclear whether their effects on mental health are related to their effects on neural plasticity. We wondered whether the plasticity-promoting effects of hallucinogens could slow or reverse the synaptic disruption and the resulting sensory and behavioral deficits in Alzheimer's. Here we tested this idea by using ketamine in a mouse model of Alzheimer's.

There are at least four hypotheses for how ketamine might be therapeutic for Alzheimer's, which are not mutually exclusive. First, the plasticity hypothesis is based on the synaptogenic and dendritogenic effects of ketamine, which could compensate for synaptic dysfunction in Alzheimer's. Because of these plasticity-promoting effects, ketamine and other hallucinogens have been recently characterized as psychoplastogens, a class of small molecules that rapidly promote plasticity following a single administration (Olson, 2018; Vargas et al., 2021; Pryazhnikov et al., 2018; but see Li et al., 2022). These effects may be due to ketamine's activation of the mammalian target of rapamycin (mTOR) pathway, which is involved in cell growth, autophagy, and synapse formation (Jaworski and Sheng, 2006; Hoeffer and Klann, 2010; Li et al., 2010; Phoumthipphavong et al., 2016; Aleksandrova and Phillips, 2021) but is inhibited in Alzheimer's transgenic mice and by Aß_42_ (Ma et al., 2010). Second, the NMDA hypothesis is based on the hyperactivation of NMDA receptors by increased levels of soluble Aß_42_ (Reisberg et al., 2003; Bezprozvanny and Mattson, 2008; Zhang et al., 2016), which contributes to excitotoxicity and synaptic dysfunction in Alzheimer's. As an NMDA antagonist, ketamine may counteract these effects and slow the progression of Alzheimer's (Li et al., 2011), as seen with the NMDA antagonist memantine. Third, the amyloid clearance hypothesis is based on the idea that activation of mTOR in microglia could promote uptake and proteolytic degradation of Aß_42_, reducing amyloid burden and improving cognitive function (Bamberger et al., 2003; Koenigsknecht and Landreth, 2004; Mandrekar et al., 2009; Li et al., 2010; Jiang et al., 2014; Price et al., 2020; Companys-Alemany et al., 2022; Shi et al., 2022). Finally, the inflammation hypothesis is based on the hallmark role of inflammation in Alzheimer's and the activation of microglia by Aß_42_, which leads to the release of proinflammatory cytokines. Ketamine reduces the levels of many of these proinflammatory factors implicated in Alzheimer's pathogenesis (Yang et al., 2013; Wang et al., 2015, 2019; Zhu et al., 2015; Tan et al., 2017; Xie et al., 2017; Chun et al., 2018), suggesting that ketamine might play an anti-inflammatory role.

These different potential mechanisms suggest quite different ketamine dosage protocols. Spinogenesis was observed within hours of a single dose, with new spines persisting for at least 2 weeks (Li et al., 2010; Phoumthipphavong et al., 2016; Moda-Sava et al., 2019). Based on these dynamics, we used a single dose of ketamine and tested for behavioral effects for 2 weeks. On the other hand, the prevention or clearance of amyloid plaques, or the prevention of NMDA-mediated excitotoxicity, might be expected to require continuous dosing over a longer timeframe [for example, Companys-Alemany et al. (2022) used a 4-week continuous dosing regimen]. For this reason, we also used a chronic daily dosing protocol with periodic behavioral testing for 16 weeks.

Our behavioral assay is auditory gap detection, a measure of temporal acuity that is an early biomarker for Alzheimer's in both mice and humans (Iliadou et al., 2017; Kaylegian et al., 2019). In this test, the detection of a brief gap in continuous white noise attenuates the startle response elicited by a loud sound. We previously saw gap detection deficits as early as 2 months of age in 5XFAD mice, a widely-used Alzheimer's model that co-expresses five familial Alzheimer's mutations and shows rapid intracellular and extracellular accumulation of Aß_42_ (Oakley et al., 2006; Kaylegian et al., 2019). This early deficit corresponds to an age at which plaque deposition has only just begun (Eimer and Vassar, 2013; Weible and Wehr, 2022), precedes evidence of structural pathology by 1–4 months (Buskila et al., 2013; Crowe and Ellis-Davies, 2013, 2014) and is at least 4 months before cell death occurs (Jawhar et al., 2012; Eimer and Vassar, 2013). Importantly, this also 2–4 months before the appearance of cognitive and memory impairments associated with hippocampal dysfunction in 5XFAD mice (Oakley et al., 2006; Ohno et al., 2006; Ohno, 2009; Devi et al., 2010; Jawhar et al., 2012; O'Leary and Brown, 2022). We hypothesized that treatments that effectively counteract the effects of increased Aß_42_ levels should positively impact behavioral gap detection.

Here we examined the effects of two different sub-anesthetic dosing protocols on behavioral gap detection in 5XFAD mice. With the “acute” dosing protocol, we tested whether a single dose of ketamine on postnatal day 119 (p119) could rescue already measurable deficits in gap detection over the following 15-day period. With the “chronic” dosing protocol, we tested whether daily injections beginning at p45 could slow or prevent the appearance of gap detection deficits over the following 111-day period. Afterward, we measured plaque load in two brain regions relevant to gap detection, primary auditory cortex and the caudal pontine reticular nucleus. We observed a robust facilitatory effect of ketamine on startle amplitude specifically in 5XFAD mice following chronic dosing but not acute dosing. No effect of ketamine was observed on gap detection following either protocol. We found strong correlations between plaque load and gap detection deficits, but ketamine had no effect on the strength of these correlations, nor any effect on plaque load. We conclude that ketamine has no effect on the development of behavioral gap detection deficits or plaque load in this Alzheimer's mouse model, following either an acute single dose or a chronic daily dose regimen.

## Methods

All procedures were performed in accordance with the National Institutes of Health guidelines, as approved by the University of Oregon Animal Care and Use Committee.

### Mice

Mice in the present study were heterozygous for 5XFAD (*n* = 27 mice, seven females and six males in the acute protocol, six females and eight males in the chronic protocol; stock number 006554, The Jackson Laboratory) on a B6SJLF1/J hybrid background (stock number 100012, The Jackson Laboratory), or wild-type (WT) B6SJLF1/J littermates as controls (*n* = 35 mice, nine females and seven males in the acute protocol, eight females and 11 males in the chronic protocol). This background is heterozygous for the retinal degeneration mutation Pde6b^rd1^, which causes blindness in Pde6b^rd1^ +/+ mice, as well as the age-related hearing loss 1 allele Cdh23^ahl^, which increases susceptibility to hearing loss in Cdh23^ahl^ −/− mice. Offspring +/+ for Pde6b^rd1^ or −/− for Cdh23^ahl^ were excluded from this study.

### Behavioral data acquisition and stimulus delivery

All data were collected in a sound-attenuating chamber. Sounds were delivered from a free-field speaker calibrated to within ±1 dB using a Brüel and Kjær 4939 1/4-inch microphone positioned where the ear would be but without the animal present. Mice were loosely restrained in a plastic tube (35 mm inner diameter, 1.5 mm wall thickness) affixed to a flat base. The tube was perforated (~3 mm diameter) to allow effective transmission of sound, with no more than 5 dB attenuation. To measure the startle response, the tube rested on a piezo transducer. Movement signals from the piezo transducer were amplified and digitized at 10 kHz.

We inserted silent gaps into continuous 80 dB background white noise and measured how these gaps attenuated startle responses elicited by a 100 dB, 25 ms white noise burst. A 50 ms interval separated the end of the gap from the start of the startle stimulus. Startle responses were measured following presentations of 4, 32, or 256 ms duration gaps, as well as to startle stimuli not preceded by a gap (“pure startle” trials) to provide a baseline startle response. Startle stimuli were presented every 12 ± 3 s. Each session included 40 presentations of each stimulus condition.

### Dosing protocols

**Figure 1:**
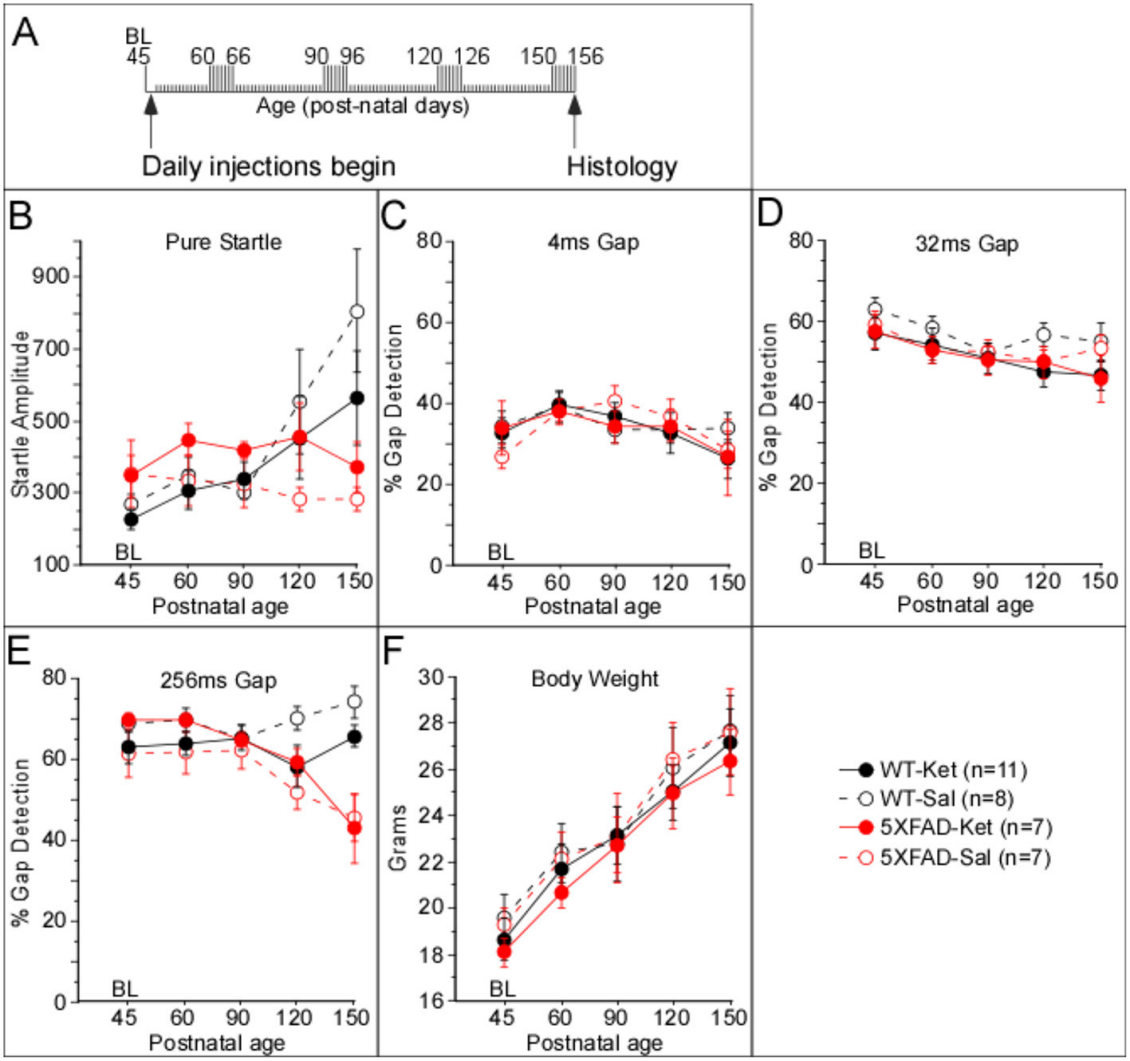
Chronic ketamine treatment affected startle amplitude, but not gap detection, in 5XFAD mice. **(A)** The timeline illustrates that daily injections of either ketamine (Ket) or saline (Sal) began after collection of baseline data on p45. Individual tick marks correspond to days, starting at p45. Tall tick marks correspond to days during which behavioral data were collected. **(B)** Startle amplitudes were robustly greater for 5XFAD mice dosed with ketamine (5XFAD-Ket) compared with 5XFAD-Sal mice, while those of WT-Ket mice were modestly but significantly smaller than those of WT-Sal mice. No effects for group, treatment, or genotype were observed for 4ms **(C)** and 32ms **(D)** gap detection. **(E)** A significant group effect for 256ms gap detection was observed that was driven predominantly by an age-related decline in 5XFAD performance. **(F)** No effects for group, treatment, or genotype were observed for body weight. Statistics for all comparisons are shown in Table 1. Black: wild-type (WT); red: 5XFAD; dashed lines & open circles: saline (Sal) injected mice; solid lines & filled circles: ketamine (Ket) injected mice. Data plotted as mean ± SE.

We assessed the effects of ketamine administration on pure startle amplitude and gap detection using two dosing protocols: acute and chronic. For both protocols, 5XFAD and WT mice received i.p. injections of either a sub-anesthetic dose of ketamine HCL (10 mg/kg) or saline. For both protocols, all mice received a single acclimation session to the apparatus and stimuli 1 day prior to baseline data collection. The acute dosing protocol (**Figure 3A**) was performed to determine whether ketamine would rescue gap detection at an age when deficits were already expected based on previous studies (Kaylegian et al., 2019; Weible et al., 2020). For the acute dosing protocol, baseline gap detection data were collected on p119, immediately after which mice were injected with either ketamine or saline. Gap detection was then tested for an additional 7 days from p120–p134 (1, 2, 3, 5, 7, 10, and 15 days post-injection). The chronic dosing protocol (Figure 1A) was performed to determine whether and how daily ketamine administration altered gap detection, startle responses, and plaque deposition relative to saline-injected controls. For the chronic dosing protocol, baseline gap detection data were collected for seven consecutive days starting on day p45, with four periods of additional data collected for seven consecutive days beginning at each of p60, p90, p120, and p150. Daily injections of sub-anesthetic ketamine or saline began following the p45 session and continued daily through p156 (injections were administered within 2 h following the end of the session).

### Histology

#### Sectioning

Within 24 h of the final gap detection session, all mice were perfused transcardially with 0.1M phosphate-buffered saline (PBS) followed by 4% paraformaldehyde in PBS (60 mL, 2 mL/min). Brains were removed and post-fixed for an additional 24 h in 4% paraformaldehyde, then sectioned or transferred to PBS containing 0.01% sodium azide. Sections (30 μm, coronal plane) including auditory cortex and the caudal pontine reticular nucleus were collected in PBS.

#### Immunohistochemistry

Free-floating sections were washed in PBS with 0.4% Triton and then blocked in 3% normal goat serum (Abcam; ab7481) for 1 h. Sections were then incubated with rabbit monoclonal anti-Aß42 (1:1,000; Invitrogen; 700254) primary antibody at 4°C on a shaker for 22 h. Sections were then rinsed thoroughly (3 × 5 min) in PBS with 0.4% Triton and incubated with AlexaFluor 488 secondary antibody (1:1,000; ThermoFisher; A-11034) on a shaker for 2 h at room temperature. Following a second thorough rinse in PBS, sections were mounted on charged slides, allowed to dry completely, and then coverslipped with DAPI Fluoromount-G (SouthernBiotech; 0100-20).

#### Imaging, boundary determination, and quantification

As described previously (Weible and Wehr, 2022), photomicrographs were taken using a Zeiss Imager A.2 microscope with AxioCam MRm camera and X-Cite 120Q light source and captured using Zen 3.1 software. Low magnification images (12.5X–50X) were taken for alignment with sections in the Allen Mouse Brain Common Coordinate Framework (CCFv3) online atlas (https://scalablebrainatlas.incf.org/mouse/ABA_v3) as described below. High magnification (200X) images were taken to quantify the number and mean area of antibody-labeled plaques. Images were captured at multiple focal depths to ensure accurate boundaries of identified plaques. Plaque counting and boundary assignment were performed manually using Canvas X software (ACD Systems). We observed two types of plaques: dense core plaques and diffuse aggregations of extracellular Aß_42_. We did not attempt to differentiate between dense core and diffuse plaques, instead counting both types together simply as “plaques.” Quantification was performed blind to genotype, sex, and injection type.

Following the identification of all plaques within the field of view, boundaries for the region of interest were applied. Boundaries, including subdivisions, were obtained by aligning sections to the CCFv3 atlas using an affine transformation based on local landmarks visible in DAPI (blue) and antibody (green) fluorescence channels. For primary auditory cortex, laminar boundaries were determined as previously described (Anderson et al., 2009; Weible and Wehr, 2022). Briefly, layers 1, 2, 3, 4, 5a, 5b, 6a, and 6b each represent 12.5% of the cortical thickness from the pial surface. The area of each region of interest was calculated by calibrating image size using a stage micrometer (2 mm-200 division KR867, MicroscopeWorld).

### Data analysis

For behavioral sessions, we quantified startle amplitudes by calculating the area of the rectified signal from the piezo transducer within a 100 ms window after startle stimulus onset. We quantified gap detection as the percent reduction in the median startle response compared to the median pure startle response for each session. For both acute and chronic dosing experiments, the first behavioral session was used for acclimation to the behavioral apparatus, and not included for data analysis. Histological comparisons included plaques/mm^2^, mean plaque size (in square microns), and % plaque coverage [(sum of plaque sizes)/(area of brain region)].

### Statistics

We analyzed group data using non-parametric tests because some of the comparisons involved non-normally distributed data (Lilliefors test), and because statistical power was comparable even when the underlying assumptions for the corresponding parametric analysis were met (Kitchen, 2009). Comparisons involving a single factor (genotype, sex, treatment) were performed using the Mann–Whitney (MW) test. Comparisons involving two factors (e.g., genotype and treatment) or a single factor with more than two variables (e.g., cortical layers or gap durations) were performed using the Kruskal-Wallis (KW) test, and are reported as “Group” tests in Tables 1–4. *Post-hoc* analyses for these KW tests were then performed using the MW with a Bonferroni correction for multiple comparisons. For the Acute dosing protocol, we used the signed-rank test (SR, a non-parametric version of the paired *t*-test) to compare behavioral gap detection during the single baseline session with the mean of the 7 post-injection days. Where appropriate to test for interactions, we employed the Scheirer-Ray-Hare (SRH) test, a non-parametric alternative to the 2-way ANOVA (Scheirer et al., 1976). We report effect sizes as eta-squared (η^2^). η^2^ varies between 0 and 1, and corresponds to the proportion of variance in the dependent variable explained by the independent variable. η^2^ values of 0.01–0.06 are generally considered small effects, η^2^ of 0.06–0.14 moderate effects, and η^2^ > 0.14 large effects. We also tested for correlations between plaque metrics and behavioral gap detection using Pearson's correlation, for which we report the significance (*p*-value) and the coefficient of determination (*r*^2^).

**Table 1:** Statistical summary of effects of chronic ketamine on gap detection behavior.

**Groups**	**Column 1**		**Column 2**		**Column 3**	
	**KW: groups**		**MW: genotype**		**MW: treatment**	
	***p*** **-value**	η^2^	***p*** **-value**	η^2^	***p*** **-value**	η^2^
**Pure startle**						
WT-Ket	**0.007**	**0.06**	0.67^**a**^	0	0.03	0.05
WT-Sal										
5XFAD-Ket									**0.003**	**0.13**
5XFAD-Sal										
**4ms gaps**						
WT-Ket	0.97	0	0.62	0	0.97	0
WT-Sal										
5XFAD-Ket									0.92	0
5XFAD-Sal										
**32ms gaps**						
WT-Ket	0.13	0.02	0.29	0.01	0.07	0.04
WT-Sal										
5XFAD-Ket									0.33	0.01
5XFAD-Sal										
**256ms gaps**						
WT-Ket	**0.0005**	**0.09**	**0.002** ^**b**^	**0.06**	**0.01**	**0.06**
WT-Sal										
5XFAD-Ket									0.10	0.04
5XFAD-Sal										

^a^SRH, p **=** 0.02, η^2^ = 0.07.

^b^SRH, p **=** 0.01, η^2^ = 0.07.

Significant effects are in bold. Within genotype tests of treatment corrected for multiple comparisons (corrected α = 0.0125). Effect size reported as η^2^.

**Table 2:** Chronic dosing summary of plaque measure analyses for deep layer auditory cortex (ACtx) and caudal pontine reticular nucleus (PRNc).

**Region**	**Column 1**		**Column 2**		**Column 3**	
	**KW: group**		**MW: treatment**		**MW: sex**	
	***p*** **-value**	η^2^	***p*** **-value**	η^2^	***p*** **-value**	η^2^
**% Plaque coverage**						
ACtx	0.71	0	0.73	0.01	0.30	0.07
PRNc	0.96	0	0.64	0.01	0.82	0
**Plaques/mm** ^2^						
ACtx	0.78	0	0.73	0.03	0.35	0.06
PRNc	0.75	0	0.60	0.02	0.86	0
**Mean plaque size**						
ACtx	0.50	0	0.64	0	0.17	0.02
PRNc	**0.04**	**0.47**	**0.04**	**0.29**	0.42	0.04

Significant effects are in bold. Effect size reported as η^2^.

**Table 3:** Statistical summary of effects of acute ketamine on gap detection behavior.

**Groups**	**Column 1**		**Column 2**		**Column 3**	
	**KW: groups**		**MW: genotype**		**SR: treatment**	
	***p*** **-value**	η^2^	***p*** **-value**	η^2^	***p*** **-value**	η^2^
**Pure startle**						
WT-Ket	0.13	0.05	0.08	0.05	0.61	0.22
WT-Sal					0.11	0.53
5XFAD-Ket					0.74	0.13
5XFAD-Sal					0.25	0.47
**4ms gaps**						
WT-Ket	0.52	0.01	0.51	0.01	**0.02**	**0.89**
WT-Sal					**0.03**	**0.73**
5XFAD-Ket					0.13	0.20
5XFAD-Sal					**< 0.05**	**0.81**
**32ms gaps**						
WT-Ket	0.22	0.03	** < 0.05**	**0.07**	0.50	0.25
WT-Sal					0.86	0.06
5XFAD-Ket					0.50	0.26
5XFAD-Sal					0.92	0.04
**256ms gaps**						
WT-Ket	**0.004**	**0.19**	**0.0004**	**0.22**	0.87	0.06
WT-Sal					0.31	0.34
5XFAD-Ket					0.61	0.19
5XFAD-Sal					0.92	0.04

Significant effects are in bold. Effect size reported as η^2^.

**Table 4:** Acute dosing summary of plaque measure analyses for deep layer auditory cortex (ACtx) and caudal pontine reticular nucleus (PRNc).

**Region**	**Column 1**		**Column 2**		**Column 3**	

	**KW: group**		**MW: treatment**		**MW: sex**	
	***p*** **-value**	η^2^	***p*** **-value**	η^2^	***p*** **-value**	η^2^
**% Plaque coverage**						
ACtx	**0.04**	**0.63**	0.42	0.05	**0.007**	**0.62**
PRNc	0.58	0	0.52	0.03	0.34	0.08
**Plaques/mm** ^2^						
ACtx	**0.04**	**0.67**	0.34	0.08	**0.007**	**0.62**
PRNc	0.59	0	0.87	0	0.26	0.11
**Mean plaque size**						
ACtx	0.76	0	0.52	0.03	0.42	0.05
PRNc	0.27	0.11	0.26	0.11	0.11	0.21

Significant effects are in bold. Effect size reported as η^2^.

## Results

Previous work has shown progressive behavioral gap detection deficits and Aß_42_ pathology as 5XFAD mice age (Kaylegian et al., 2019; Weible and Wehr, 2022). Here we hypothesized that low-dose ketamine would improve behavioral gap detection, and that this might be explained by a reduction in plaque load in two regions critically involved in gap detection: primary auditory cortex and/or the caudal pontine reticular nucleus. We tested for these effects in two groups of mice, one receiving daily injections of low-dose ketamine beginning at p45 (Chronic Dosing Protocol) and another receiving a single dose at 4 months of age (Acute Dosing Protocol). While ketamine did not rescue behavioral gap detection following either protocol, we did observe some effects of ketamine on both behavior and pathology. We also report for the first time a direct correlation between gap detection deficits and plaque load.

### Chronic dosing protocol

#### Effects of chronic ketamine on gap detection behavior

To assess the impact of chronic, sub-anesthetic ketamine administration on behavior, we collected baseline data at p45, after which 5XFAD and wild-type (WT) littermate mice began receiving daily injections of either ketamine (Ket) or saline (Sal). We then tested behavior daily for 7 days beginning at p60, and again at p90, p120, and p150 (Figure 1A). We observed a significant difference among these four groups for pure startle amplitude (Figure 1B; Table 1, Column 1). Startles were significantly more robust for 5XFAD-Ket vs. 5XFAD-Sal mice (Table 1, Column 3). The strength of this effect, coupled with the overlap at baseline (closed vs. open red circles at p45 in Figure 1B), indicates that this is a genuine effect of treatment. In contrast, WT-Ket mice appeared to have *smaller* startles compared with WT-Sal mice, though this difference was not significant when corrected for multiple comparisons (Table 1, Column 3). Startles for 5XFAD-Ket mice were initially larger than, but followed the same trajectory as, those of both WT-Ket and WT-Sal mice from p45 to p90. From p90 on they plateaued, while those of WT mice continued to increase with age. While there was no main effect of genotype on startle responses, there was a significant interaction between genotype and age (Table 1, Column 2, SRH test), reflecting an increase in WT startles with age that was absent in 5XFAD mice. Thus 5XFAD mice that received chronic saline injections and behavioral testing showed a progressive reduction of startle responses (consistent with previous findings in Weible et al., 2020), but this reduction was not seen in mice that received chronic ketamine injections. As described below, we did not observe this with the acute protocol.

For gap detection, mice showed no treatment or genotype differences for shorter gaps (4 and 32 ms; Figures 1C, D; Table 1, Column 1 ). We did observe a significant effect among the four groups for 256ms gap detection (Figure 1E; Table 1, Column 1). In contrast to the effect on pure startle amplitudes, however, this did not appear to be attributable to ketamine treatment. 5XFAD mice exhibited impaired 256ms gap detection that worsened with age relative to WTs (Table 1, Column 2, SRH test; consistent with previous findings in Weible et al., 2020). While a significant effect of treatment was seen with WT mice (Table 1, Column 3, with correction for multiple comparisons), this is likely simply a cohort difference between Ket and Sal injected mice, as the two groups already differed at baseline (black closed and open circles at p45) and both WT groups followed a similar trajectory with postnatal age. Thus ketamine did not appear to have any effect on gap detection deficits in 5XFAD mice.

We measured startle responses (and thus gap detection) using a pressure sensor, which is sensitive to body weight. We therefore tested whether differences in weight among groups over time could potentially account for any of the effects we observed. No differences in weight were observed among the four groups, or when data were analyzed by genotype or treatment (Figure 1F).

#### Effects of chronic ketamine on plaque load

**Figure 2:**
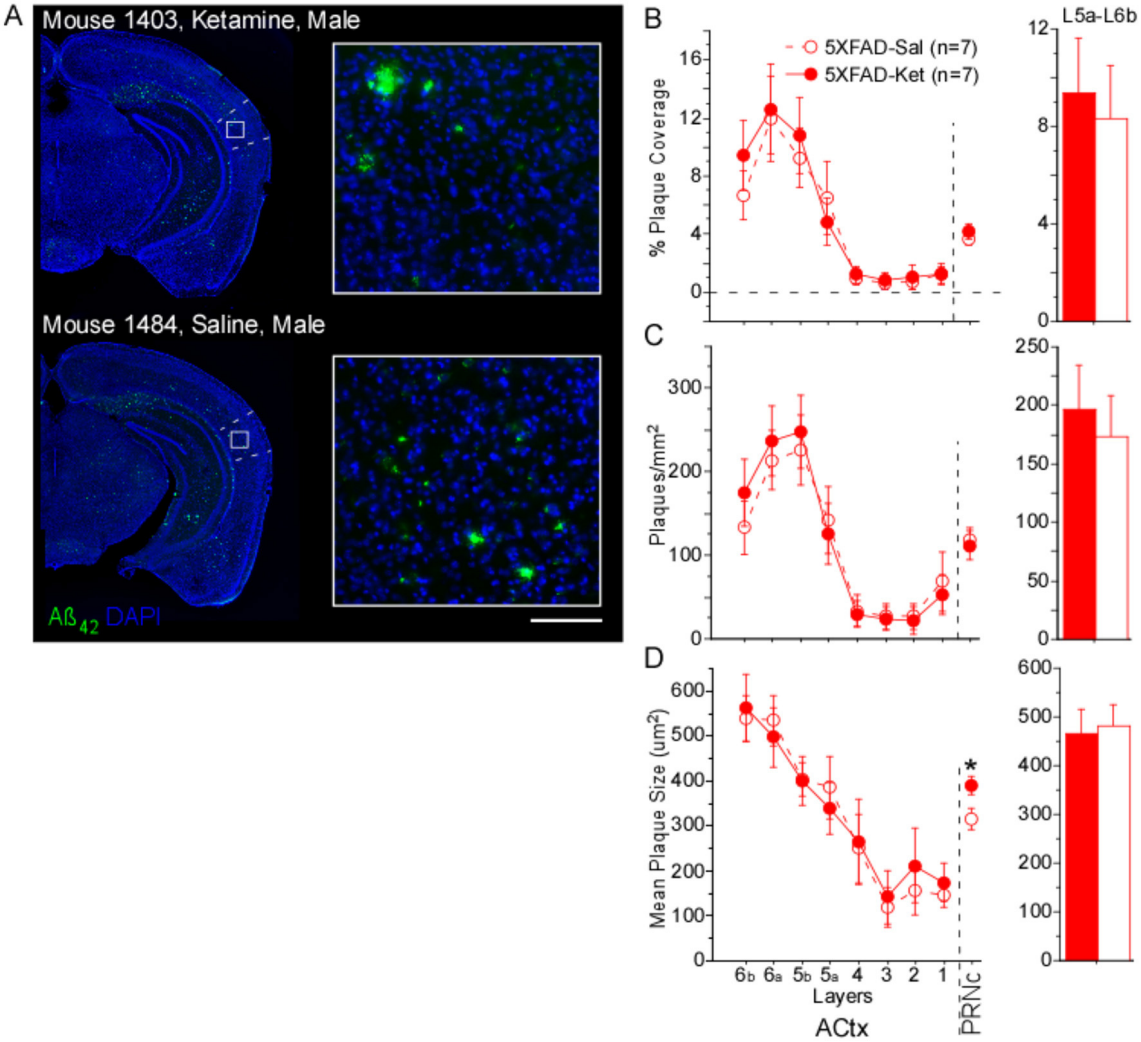
Chronic ketamine dosing did not alter plaque metrics in primary auditory cortex (ACtx), but increased mean plaque size in caudal pontine reticular nucleus (PRNc). **(A)** Representative brain sections from a ketamine-treated mouse and a saline-treated mouse (both 5XFAD, age p156). Green: anti-Aβ_42_ antibody label. Blue: DAPI-labeled nuclei. Dashed lines indicate borders of primary auditory cortex, white box indicates location of high magnification insets at right. Scale bar in inset is 100 μm. No antibody labeling was observed in sections from WT mice. **(B)** % Plaque coverage, **(C)** plaques/mm^2^, and **(D)** mean plaque size were quantified across layers in primary auditory cortex (ACtx), as well as in PRNc. In PRNc, a significant treatment effect was seen for mean plaque size, with larger plaques observed in ketamine-treated compared with saline-treated 5XFAD mice. No effects of treatment were observed in deep layers 5a-6b of ACtx. Statistics for all comparisons are shown in Table 2. Dashed lines & open circles: saline (Sal) injected mice. Solid lines & filled circles: ketamine (Ket) injected mice. Data plotted as mean ± SE. *p **<** 0.05.

To determine whether chronic ketamine treatment had an effect on amyloid plaque load, we compared plaque coverage (in %), plaque density (in plaques/mm^2^), and mean plaque size in two brain regions known to be involved in gap detection: primary auditory cortex (ACtx) and the caudal pontine reticular nucleus (PRNc). As WT mice had no amyloid plaques, we only examined the effects of ketamine in 5XFAD mice. Ketamine had almost no effect on Aß_42_ plaque measures (Figure 2). The only significant effect of ketamine we saw was an increase in mean plaque size in PRNc (Figure 2D; Table 2, Column 1). In the superficial layers of ACtx, labeling of plaques was very sparse, consistent with previous work (Weible and Wehr, 2022). Based on this, we wondered if the lack of any significant effect of ketamine in ACtx could be due to a floor effect in the superficial layers, and so we examined plaque measures specifically in deep layers 5a−6b. However, even in the deep layers of ACtx, we saw no significant effects among the four groups (male and female mice injected either with ketamine or saline), or when analyzing by treatment or sex for any of the plaque measures. As noted above, we observed no antibody label for Aß_42_ in WT mice. Thus ketamine had no effect on amyloid pathology in auditory cortex, but did specifically increase plaque size in PRNc (which is the opposite of a beneficial effect of ketamine).

### Acute dosing protocol

#### Effects of acute ketamine on gap detection behavior

**Figure 3:**
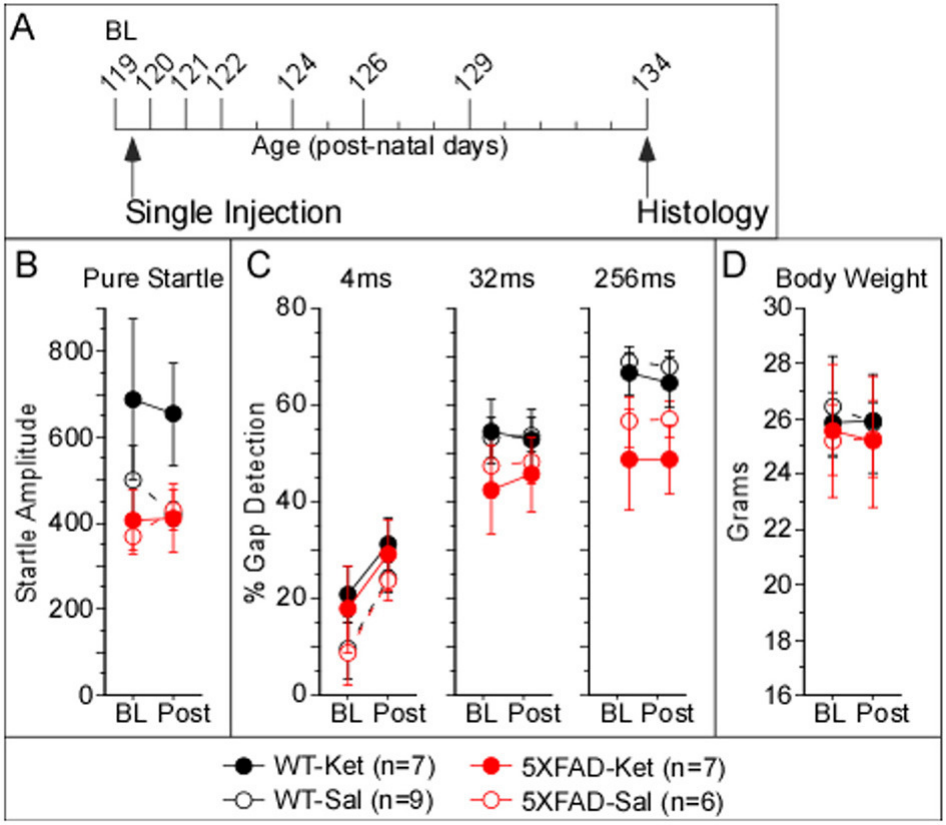
Acute ketamine treatment at p120 had no impact on behavioral gap detection over the following 2 weeks. **(A)** Acute dosage and testing protocol. Individual tick marks correspond to days, and tall ticks indicate days of behavioral gap detection testing. Mice received a single dose of ketamine after baseline testing at age p119, followed by 7 days of testing from ages p120–p134, after which brains were processed for Aß_42_-antibody labeling. Acute dosing with ketamine appeared to have no effect on **(B)** pure startle amplitudes, **(C)** gap detection, or **(D)** body weight. Statistics for all comparisons are shown in Table 3. Data plotted as mean ± SE.

To determine whether a single sub-anesthetic dose of ketamine would impact gap detection in 5XFAD mice, we compared baseline data collected at p119 to mean post-injection behavioral performance collected for 7 days over the following 2 weeks (Figure 3A).

We found no significant effects on pure startle amplitude following acute dosing with ketamine or saline (Figure 3B, Table 3). A significant difference among 5XFAD-Ket, 5XFAD-Sal, WT-Ket, and WT-Sal groups of mice for 256ms gap detection, and a trend for 32ms gap detection, (Figure 3C, Table 3, Column 1) were attributable to effects of genotype (Table 3, Column 2), as no group showed a significant change between baseline and the post-injection interval. Signed-rank comparisons of baseline and post-injection means did reveal effects for 4ms gap detection (Table 3, Column 3). However, these effects were seen following both saline and ketamine injections, and thus appear to reflect an improvement in detection of the briefest gap across sessions rather than an effect of treatment. Body weight showed no differences across groups, genotypes, or treatments (Figure 3D).

#### Acute plaque load

**Figure 4:**
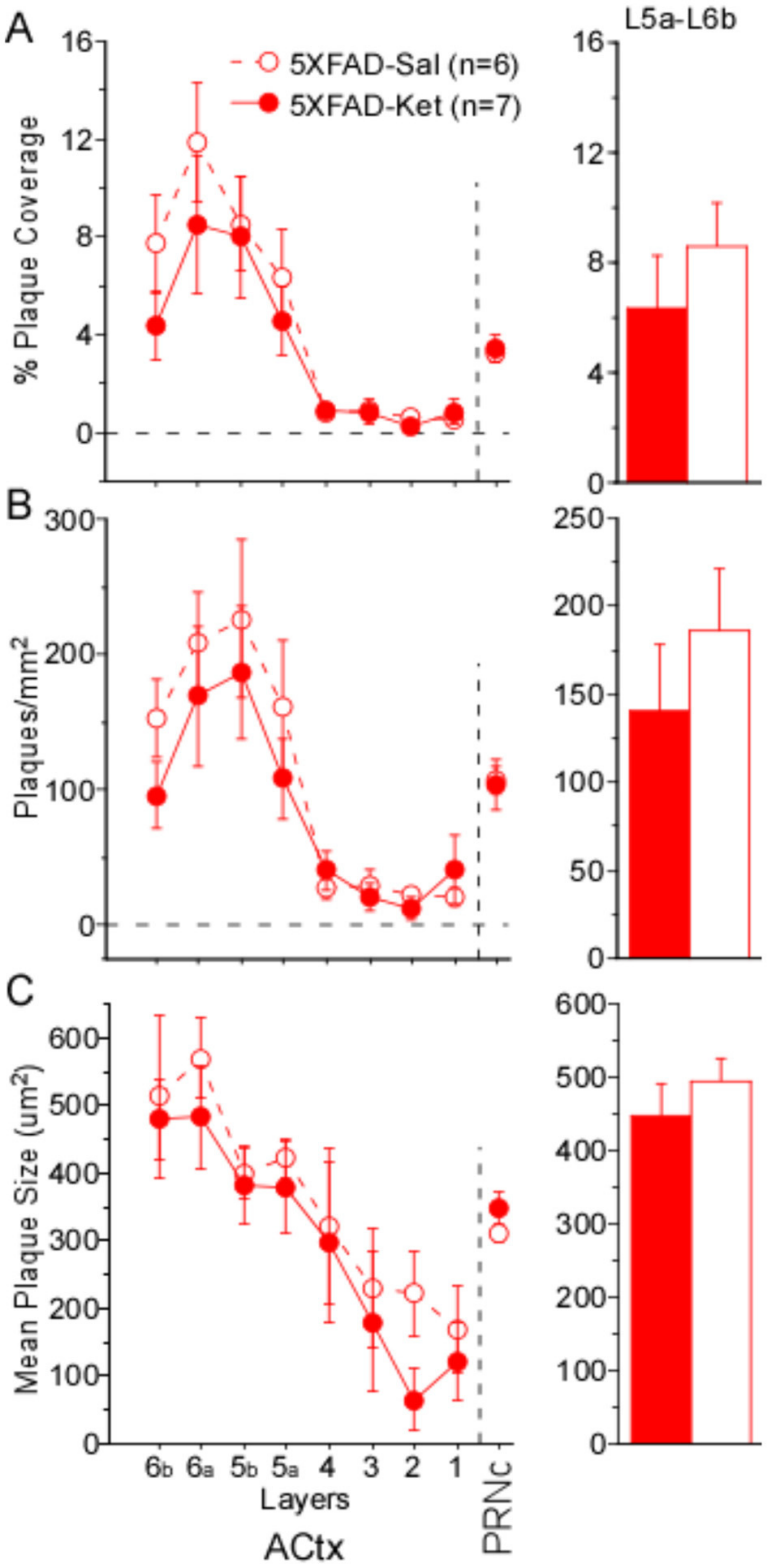
A single, acute dose of ketamine at postnatal day 119 did not significantly alter plaque load 2 weeks later. **(A)** % Plaque coverage, **(B)** plaques/mm^2^, and **(C)** mean plaque size were quantified across layers in primary auditory cortex (ACtx), as well as in the caudal pontine reticular nucleus (PRNc). No significant effects of treatment were observed in deep layers 5a–6b of ACtx or in PRNc. Statistics for all comparisons are shown in Table 4. Dashed lines and open circles: saline (Sal) injected mice. Solid lines & filled circles: ketamine (Ket) injected mice. Data plotted as mean ± SE.

To determine whether acute ketamine treatment had an effect on plaque load, we compared % plaque coverage, plaques/mm^2^, and mean plaque size in ACtx and PRNc of male and female 5XFAD-Ket and 5XFAD-Sal mice (Figures 4A–C, respectively). As described above for chronic dosing, our quantification of cortical plaques following acute dosing focused on deep layers 5a−6b. In cortex, significant effects among the four groups (treatment X sex) were observed for % plaque coverage and plaques/mm^2^ (Table 4, Column 1). These effects were driven predominantly by robust sex effects, with significantly greater plaque coverage and density observed in females (Table 4, Column 3). Although Figures 4A, B do appear to show a slight beneficial effect of ketamine treatment, which lowered plaque coverage and plaques/mm^2^, these effects were not significant and the effect sizes were small (Table 4: η^2^ = 0.05 and η^2^ = 0.08). A power analysis indicated that for an effect this small to achieve significance, we would need to roughly quintuple our sample size (from *n* = 13 to *n* = 62 mice). Thus we cannot rule out the possibility that acute ketamine might have a slight beneficial effect on plaque load, but our data indicate that any such effect is likely to be negligible. We found no effects of group, treatment, or sex on any plaque measures in PRNc. We observed no antibody label for Aß_42_ in WT mice.

### Protocol effects on behavior

**Figure 5:**
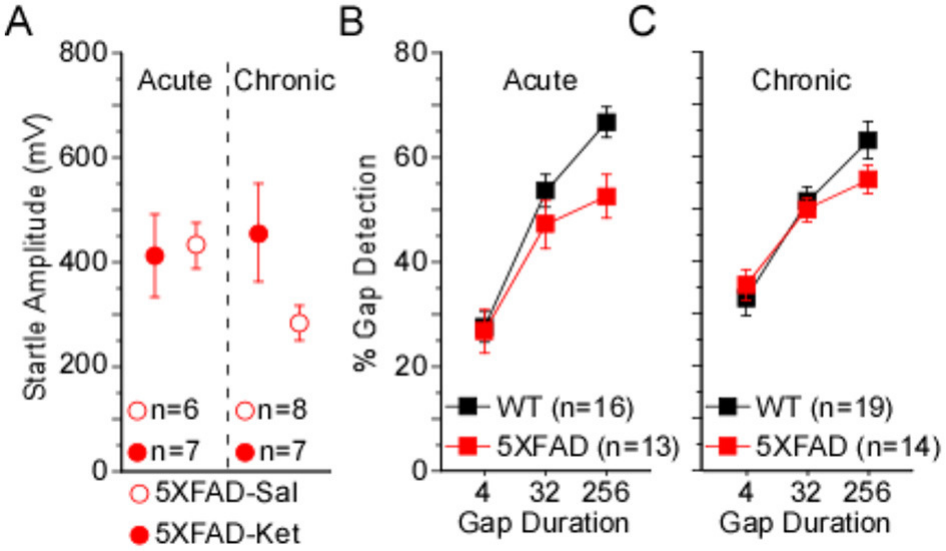
Protocol differences influence behavior when measured at p120. **(A)** In the chronic dosing protocol (see Figure 1), 5XFAD-Sal mice exhibited significantly smaller startles compared with 5XFAD-Ket mice. At p120, chronic ketamine dosing in 5XFAD mice resulted in startles comparable to those of acute 5XFAD-Sal and 5XFAD-Ket mice. **(B, C)** Detection of 4ms gaps was significantly greater for chronic mice compared with acute mice. An effect of genotype also trended toward significance for acute mice, but not chronic mice. Open red circles: saline injected 5XFAD mice (5XFAD-Sal); closed red circles: ketamine injected 5XFAD mice (5XFAD-Ket); black squares: wild-type (WT); red squares: 5XFAD. Data plotted as mean ± SE.

The chronic dosing protocol had far more repeated behavioral testing and animal handling than the acute protocol, which could lead to experience-dependent differences in behavior. To assess this, we compared behavior from chronic mice and acute mice, both at p120, and found two notable differences. First, chronic 5XFAD-Ket mice had significantly greater startles relative to chronic 5XFAD-Sal mice (see Figure 1B). At p120, this effect is robust (η^2^ = 0.18). In contrast, ketamine had no effect on startles in the acute protocol (see Figure 3B). A side-by-side comparison (Figure 5A) illustrates that chronic 5XFAD-Ket startles were comparable to those of both 5XFAD-Ket *and* 5XFAD-Sal acute mice. Thus, it is not simply that startles are reduced in older 5XFAD mice. Instead, we conclude that the cumulative impact of daily handling, daily injections, and (by p120) sixteen previous gap detection sessions resulted in a reduction in startle responses which was reversed by chronic ketamine dosing specifically in 5XFAD mice. The second notable difference was with gap detection itself. As ketamine did not impact gap detection in either dosing protocol, we combined treatments and compared gap detection by protocol and genotype. Chronic mice exhibited significantly better 4ms gap detection compared with acute mice (Figure 5B vs. Figure 5C; MW: *p* = 0.03, η^2^ = 0.07). This is likely the same effect as the experience-related improvement in 4ms gap detection seen for all acute groups in Figure 3C. Taken together, these experience-dependent effects don't alter our conclusions about the effects of ketamine on 5XFAD mice, but are important to keep in mind when comparing the effects of the two dosing protocols.

### Behavior correlates with plaque load

**Figure 6:**
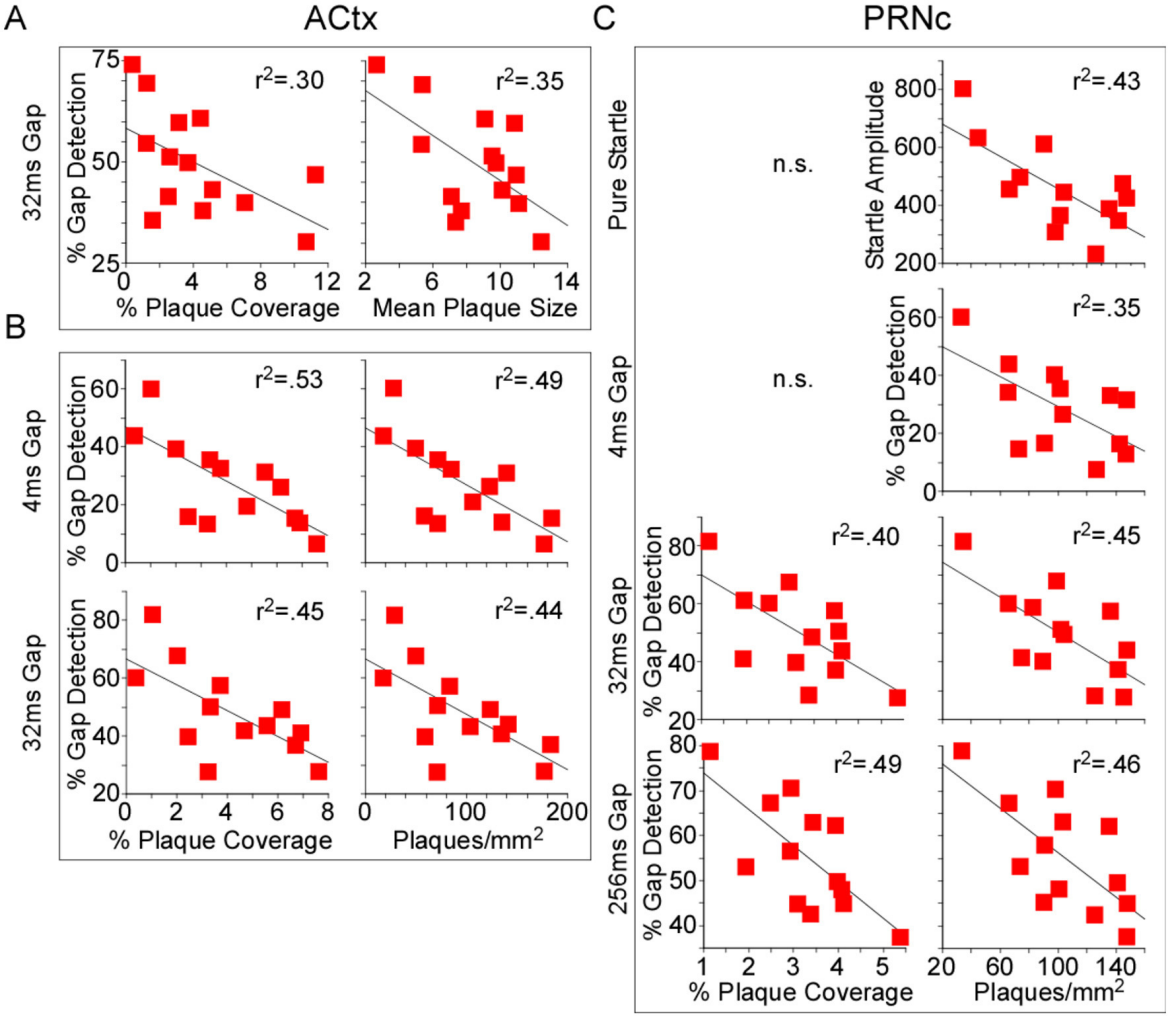
Behavioral gap detection correlated inversely with plaque load. Because no significant effects of treatment were observed, we pooled 5XFAD-Ket and 5XFAD-Sal mice (red squares) and tested for correlations (Pearson) between plaque load metrics and behavior. **(A)** In auditory cortex (ACtx) of p150 chronic mice, 32ms gap detection decreased as both % plaque coverage and mean plaque size increased. **(B)** In ACtx of p120 acute mice, 4ms and 32ms gap detection decreased as % plaque coverage and plaques/mm^2^ increased. **(C)** In the caudal pontine reticular nucleus (PRNc) of these same mice, 32ms and 256ms gap detection decreased as % plaque coverage increased, and plaques/mm^2^ correlated inversely with startle amplitude and each gap duration tested. Statistics for all comparisons are shown in Table 5. *r*^2^: square of the Pearson correlation coefficient. n.s.: not significant.

In this study we measured both gap detection deficits and amyloid pathology in a large cohort of mice, allowing us for the first time to directly test whether the degree of gap detection deficits were correlated with the extent of amyloid pathology across mice. We found that they were. Because we saw no significant effects of treatment on gap detection, we pooled 5XFAD-Ket and 5XFAD-Sal mice and tested for correlations between behavior and each of our plaque measures. For chronic mice (p150), 32ms gap detection decreased as both % plaque coverage and mean plaque size in ACtx increased (Figure 6A, Table 5). For acute mice (p120), both 4ms and 32ms gap detection decreased as % plaque coverage and plaques/mm^2^ in ACtx increased (Figure 6B, Table 5). In PRNc, plaque metrics were similarly correlated with gap detection, but only in acute mice. In these mice, % plaque coverage correlated inversely with 32ms and 256ms gap detection, and plaques/mm^2^ increased as each of the behavioral measures decreased (Figure 6C, Table 5). Thus gap detection deficits in 5XFAD mice are correlated with the degree of amyloid pathology in both auditory cortex and PRNc, confirming previous work demonstrating that these brain regions contribute to gap detection and showing that the behavioral deficits seen in 5XFAD mice are directly related to amyloid accumulation in these brain regions.

**Table 5:** Statistical summary of behavior/plaque measure correlations.

	**% Plaque coverage**		**Plaques/mm** ^**2**^		**Mean plaque size**	

	***p*** **-value**	*r* ^2^	***p*** **-value**	*r* ^2^	***p*** **-value**	*r* ^2^
**Acute: primary auditory cortex**						
Pure startle	0.10	0.25	0.08	0.28	0.90	0
4ms gap	**0.005**	**0.53**	**0.01**	**0.49**	0.51	0.05
32ms gap	**0.02**	**0.45**	**0.02**	**0.44**	0.68	0.02
256ms gap	0.09	0.27	0.08	0.28	0.91	0
**Acute: caudal pontine reticular nucleus**						
Pure startle	0.07	0.29	**0.02**	**0.43**	0.36	0.09
4ms gap	0.11	0.24	**0.04**	**0.35**	0.24	0.14
32ms gap	**0.02**	**0.40**	**0.01**	**0.45**	0.53	0.04
256ms gap	**0.009**	**0.49**	**0.01**	**0.46**	0.85	0
**Chronic: primary auditory cortex**						
Pure startle	0.19	0.14	0.20	0.14	0.17	0.16
4ms gap	0.82	0.01	0.77	0.01	0.51	0.04
32ms gap	**0.04**	**0.30**	0.07	0.25	**0.02**	**0.35**
256ms gap	0.15	0.17	0.23	0.12	0.08	0.24
**Chronic: caudal pontine reticular nucleus**						
Pure startle	0.73	0.01	0.99	0	0.59	0.03
4ms gap	0.37	0.07	0.36	0.07	0.74	0.01
32ms gap	0.26	0.11	0.74	0.01	0.07	0.25
256ms gap	0.85	0	0.82	0.01	0.29	0.10

Significant effects are in bold.

## Discussion

Ketamine has received growing attention for its effects on neuroplasticity and neuroinflammation, and as a treatment for depression, post-traumatic stress disorder, and substance use disorder. Here we examined whether ketamine had beneficial effects on behavioral gap detection deficits and amyloid plaque formation in the 5XFAD mouse model of Alzheimer's disease. We found that chronic dosing with ketamine increased startle responses specifically in 5XFAD mice, but had no impact on gap detection behavior, and did not reduce plaque load as measured at p150. A single, acute dose of ketamine at p120 had no impact on gap detection behavior, and a negligible (not statistically significant) effect on plaque load in auditory cortex. Thus, our findings do not suggest that ketamine is effective at reducing plaque load nor for improving gap detection deficits in 5XFAD mice. However, irrespective of ketamine treatment, we did find that gap detection deficits were correlated with plaque load in auditory cortex and in the caudal pontine reticular nucleus, showing that these behavioral deficits seen in 5XFAD mice are directly related to amyloid accumulation in these two brain regions. This finding reinforces the validity of gap detection as an early biomarker of Alzheimer's (Kaylegian et al., 2019; Weible et al., 2020) that could be useful for testing the effectiveness of pharmaceutical interventions geared toward reducing plaque load.

We found that ketamine had a specific effect on pure startle responses in 5XFAD mice. Although this effect is unrelated to the gap detection deficits and amyloid pathology we hypothesized might be rescued by ketamine, it is intriguing. We and others have found that startle responses are reduced in 5XFAD mice (Bhattacharya et al., 2014; Weible et al., 2020), and also in the Tg4-42 Alzheimer's mouse model (Sichler et al., 2019). Chronic ketamine administration significantly increased startle responses in 5XFAD mice (Figure 1B), consistent with a partial rescue effect of ketamine. However, chronic ketamine treatment did not improve gap detection performance and moreover was associated with increased plaque size in the PRNc. Also, acute ketamine had no effect on startle responses. This suggests that ketamine may interact with genotype and experience-dependent effects of daily handling and injections, perhaps by affecting stress or habituation. Thus the effect of ketamine on startle responses, although specific to 5XFAD mice, likely does not reflect a rescue effect on Alzheimer's-related deficits.

Although several lines of evidence suggest that ketamine could in principle have beneficial effects on behavioral and molecular consequences of Alzheimer's disease—such as promotion of synaptic plasticity, reduction of NMDA-mediated excitotoxicity, clearance of amyloid burden, and reduction of neuroinflammation—our results suggest that none of these potential therapeutic effects were strong enough to detect with our approach. Ketamine may have slightly reduced amyloid plaques, but this effect was very small and did not reach statistical significance. We did not measure plasticity or inflammation, but even if ketamine had any effect on these factors, there was no downstream effect on gap detection behavior. In the future, it would be interesting to test for possible effects of ketamine on gliosis or on the processing and degradation of amyloid precursor protein (APP). In sum, our results provide no evidence for or against these hypothesized mechanisms by which ketamine might be therapeutic for Alzheimer's, but they do suggest that any such therapeutic effect is likely to be minimal.

Overall, our results indicate that sub-anesthetic ketamine is not an effective intervention for reversing auditory behavioral deficits or reducing plaque deposition in 5XFAD mice, at least under the treatment regimens we used. This contrasts with previous studies showing the benefits of other NMDA antagonists (such as memantine) in reducing Aβ levels and cognitive impairment (Liu et al., 2014; Wang et al., 2015; Companys-Alemany et al., 2022). These divergent effects could stem from differences in how ketamine and drugs like memantine interact with NMDA receptors. Although both are non-competitive NMDA antagonists, memantine and ketamine have different pharmacological profiles and produce strikingly different clinical and behavioral outcomes (Johnson et al., 2015; Smalheiser, 2019). For example, memantine may preferentially inhibit extrasynaptic NMDA receptors, whereas ketamine may preferentially block synaptic receptors (Johnson et al., 2015). On the other hand, a recent study found that a single acute dose of ketamine partially rescued synaptic activity and plasticity in the App^NL − F^ Alzheimer's model mouse (Portal et al., 2024). This effect was seen 24 h after ketamine injection, which was administered early in disease progression, when synaptic deficits are present but before the appearance of Aß plaques or neuronal damage. This finding is promising, and it's possible that the differences from our results arise from the different treatment protocol, the different mouse model, and/or the fact that they tested for synaptic function in hippocampal slices (among other measures), whereas we tested auditory behavior and plaque load. Portal et al. (2024) also raised the question of whether chronic ketamine administration might have a prophylactic effect on Alzheimer's symptoms; however, our results suggest that chronic administration offered no benefit over acute administration, at least for the measures we tested. Ketamine might also act via metabolites such as hydroxynorketamine (Johnson et al., 2015; Smalheiser, 2019), which could therefore be tested as an alternative to ketamine that might provide the beneficial effects of ketamine without the side effects. Indeed, Ribeiro et al. (2024) recently tested the effects of hydroxynorketamine in two Alzheimer's mouse models, and found that it induced signaling and transcriptional responses that rescued synaptic and memory deficits. Ketamine has also been proposed as a symptomatic treatment for Alzheimer's due to its antidepressant effects, because depression is a common symptom of Alzheimer's for which typical antidepressants (such as SSRIs) are largely ineffectual (Orgeta et al., 2017; Mohammad Shehata et al., 2022). Moreover, Wang et al. (2022) suggested that ketamine, even when used only for its antidepressant effects, could nevertheless be an effective treatment for Alzheimer's because improving depression is expected to be prophylactic against the development of dementia.

One limitation of our study design is that we used only a single dosage (10 mg/kg), administered either in a single acute dose or in chronic daily doses for ~4 months. These dosage protocols could certainly be suboptimal, leaving open the possibility that a different dosage or protocol might have different effects. The acute protocol—a single dose followed by 2 weeks of testing—was based on the increased spine density seen for 2 weeks following a single ketamine injection (Phoumthipphavong et al., 2016). In contrast, our chronic protocol was based on the possibility that ketamine could prevent or clear amyloid accumulation over time, slowing disease progression. Whether alternative or hybrid protocols might engage one or more of these or other mechanisms remains an open question. Another limitation is that we used only a single behavioral assay, gap detection, which has been shown to be affected early in disease progression in both Alzheimer's patients and in Alzheimer's model mice. It is certainly possible that ketamine could have a beneficial effect on any of the many other behavioral measures affected by Alzheimer's, including tests of cognition and memory.

## Data Availability

The raw data supporting the conclusions of this article will be made available by the authors, without undue reservation.
